# Phytochemical screening, antioxidant, and antimicrobial analysis of *Portulaca oleracea* seeds with in-silico molecular docking insights

**DOI:** 10.1016/j.jgeb.2025.100516

**Published:** 2025-06-18

**Authors:** Khursheed Ahmad Sheikh, Reyaz Hassan Mir, Mohammad Ovais Dar, Adil Farooq Wali, Insha Qadir, Sheeba Nazir, Mohammed Iqbal Zargar, Sirajunisa Talath, Sathvik B. Sridhar, Javedh Shareef, Mubashir Hussain Masoodi

**Affiliations:** a Pharmaceutical Chemistry Division, Department of Pharmaceutical Sciences, School of Applied Sciences and Technology, University of Kashmir, Hazratbal, Srinagar 190006 Kashmir, India; b Pharmaceutical Chemistry, School of Pharmaceutical Education and Research (SPER), Jamia Hamdard, Hamdard Nagar, New Delhi, India; c Department of Pharmaceutical Chemistry, M. M. College of Pharmacy, Maharishi Markandeshwar (Deemed to be University), Mullana, Ambala, Haryana 133207, India; d Department of Pharmaceutical Chemistry, RAK College of Pharmacy, RAK Medical and Health Science University, Ras Al Khaimah, United Arab Emirates; e Pharmaceutical Biotechnology Division Department of Pharmaceutical Sciences, School of Applied Sciences and Technology, University of Kashmir, Srinagar 190006, India; f Department of Clinical Pharmacy & Pharmacology, RAK College of Pharmacy, RAK Medical & Health Sciences University, Ras Al Khaimah, United Arab Emirates

**Keywords:** *Portulaca oleracea*, Purslane, Natural compounds, Antioxidant, Antibacterial, Antifungal antimicrobial, Drug discovery, DPPH, Total phenolic content, Total flavonoid content

## Abstract

**Background:**

*Portulaca oleracea* (PO), an annual succulent herb with global distribution, has been used medicinally since ancient times, earning the title “global panacea.”

**Aim:**

This study aimed to perform phytochemical screening, antioxidant and antimicrobial analysis of PO seeds, including GCMS analysis of methanolic extract and molecular docking for antimicrobial mechanisms.

**Materials and Methods:**

PO seeds underwent phytochemical screening and methanolic extract analysis via DPPH, NO radical scavenging, and reducing power assays. Antimicrobial activity was tested against bacteria and fungi, with GCMS identifying compounds. Molecular docking was conducted via Autodock vina, against targets beta-tubulin (5FNV) and ABC transporter (6J9W).

**Results:**

Methanolic extract showed strong antioxidant activity (IC_50_: 125.2 µg/ml for DPPH, 402.89 µg/ml for NO) and concentration-dependent reducing power. It was highly effective against *Saccharomyces cerevisiae* and *Hypochrea viridescens*. GCMS identified colchicine, n-decanoic acid, and triepoxydecane, with colchicine showing high binding affinity to protein targets.

**Conclusion:**

The methanolic extract’s potent antioxidant and antimicrobial effects, supported by colchicine’s binding affinity, validate PO’s medicinal potential, suggesting further therapeutic exploration.

## Highlights

• The *P. oleracea* seeds have a strong antioxidant and antimicrobial activity, particularly in their methanol extract.
• The antioxidant activity of methanol extract was very high at 125.2 and 402.89 µg/ml DPPH and NO IC50 respectively, along with an increasing reducing power.
• This extract also exhibited potent antibacterial and antifungal activity against *S. cerevisiae* and *H. viridescent*.
• Colchicine proved to dock well with both beta tubulin (5FNV) and ABC transporter (6J9W), suggesting antifungal action.

## 1 Introduction

Human beings have been exploring nature for disease alleviating agents since their very existence. Several significant medicinal compounds, especially those derived from plants, were discovered due to these persistent efforts. Since then, particularly from the past few decades, there has been an increasing trend for natural product research. Our current work is also an extension of the same trend. We chose the plant *Portulaca oleracea* (P.O) for it and thoroughly investigated the antioxidant and antimicrobial potential of its seeds followed by GCMS analysis and computational studies. Almost all parts of (P.O) have been the subject of investigation, except for the seeds, which have received the least attention^1^. So, our study focused on its seeds to assess their antioxidant capabilities and antimicrobial effects. There is a tremendously increasing demand for novel antioxidants and antimicrobials, for meeting many unmet serious challenges like cancers and drug resistance.

The human body undergoes several metabolic processes that generate free radicals, which are notorious for their association with various health conditions, including cancer, neurological and cardiovascular diseases, ulcerative colitis, aging and so on. To counteract the negative effects of oxidants and free radicals, the human body is endowed with sophisticated defense mechanisms. Besides, the proper consumption of dietary antioxidants can stop the free radical damage. Further, the studies show that using a mixture of antioxidants outperforms using them alone over a long period of time[2], [3], [4].

Like antioxidants, the antimicrobials have also become a hot area of research for scientists. Every other day, we come to know of a new resistant microbial species. A very potent drug becomes completely impotent after few years, paving the way for the search of newer antimicrobials. Not only is the resistance a concern but side effects and adverse drug reactions are also of major concern besides the unaffordable prices. So, there is a strong and immediate need and demand for the novel antimicrobials originating from nature because these and the products obtained from them are far safer and affordable than the synthetic drugs[5], [6]. As per WHO, there are 21,000 potential medicinal plants and a significant majority of people worldwide (80 %), rely on herbal remedies to fulfill their primary healthcare requirements. Over three-quarters of individuals worldwide depend predominantly on medicinal plants or substances derived from plants to alleviate their illnesses and ailments[7], [8]. A large percentage of all plant species, were utilized for medicinal purposes at some point in history. As per WHO estimations, plant-based drugs make up 25 % of all medications in wealthy nations like the USA, while they make up 80 % of all medications in under-developed nations like India. The biggest advantage of plant derived drugs is their synchronization with nature and also can be used across all age groups. For these reasons, herbal treatment is gaining popularity all over the world[9], [10], [11], [12], [13], [14].

*Portulaca oleracea* Linn., commonly known as Purslane, is a plant that grows annually and is succulent in nature. It has the cosmopolitan distribution and thrives best in warm climates and is found in many parts of the world, particularly in tropical and subtropical regions, including the United States. Its use as a vegetable, spice, and medicinal plant dates back to ancient Egyptian times[15], [16]. It is known for its sour taste and cooling effect on the body. *Portulaca oleracea* is also recognized by the World Health Organization as a frequently used medicinal plant due to its ability to cool the blood, stop bleeding, clear heat, and eliminate toxins, earning it the nickname Global Panacea[17], [18], [19], [20]. While the therapeutic potential of (P.O) has garnered significant attention, existing studies predominantly focus on its leaves and stems, with well-documented antioxidant and antimicrobial activities attributed to their flavonoid and polysaccharide content. However, the seeds of *Portulaca oleracea* remain understudied despite their ecological and biochemical distinctiveness. The comprehensive review by Zhou et al. [21] catalogues over 50 compounds, including flavonoids, alkaloids, and terpenoids, but seeds are scarcely mentioned, with only dopamine and noradrenaline detected at lower concentrations compared to leaves and stems[21], [22], [23]. For instance, omega-3 fatty acids like α-linolenic acid are well-documented in leaves[24], [25], yet seed-specific lipid profiles remain uncharacterized. Similarly, alkaloids such as oleraceins and cytotoxic homoisoflavonoids like portulacanones are isolated from aerial parts[26], [27], [28], while seeds are overlooked despite their ecological role as evolutionary reservoirs of defense metabolites. Pharmacological studies emphasize leaf/stem extracts for antioxidant, antimicrobial, and antidiabetic activities[29], [30], [31], [32], with only one clinical trial noting that seed powder (5 g/day) improves glycemic control in diabetic patients, albeit without mechanistic insights^33^. Traditional systems, including Ayurveda and Unani, historically utilize seeds for infections and oxidative stress, yet modern validation is lacking. For example, seeds are ground into flour in the U.S. and Australia^34^, but their bioactive potential remains unexamined. Furthermore, while the plant’s leaves are hailed as a “vegetable for long life” in Chinese medicine[35], [36], [37], [38], seeds are absent from pharmacological discussions despite their higher phenolic content suggested in preliminary screenings^39^. Despite this, systematic comparisons of seed-derived extracts using polar solvents (e.g. methanol) remain unexplored. This study hypothesizes that *Portulaca oleracea* seeds possess unique or superior antioxidant and antimicrobial properties relative to other plant parts, owing to their distinct metabolic profile shaped by ecological and reproductive pressures. By investigating methanolic extract, we aim to bridge the gap between traditional use and contemporary evidence, while expanding the pharmacological repertoire of this multifunctional species.

## 2 Materials and Methods

### 2.1 Collection of (P.O) seeds

In April 2018, we purchased four kilograms of (P.O) seeds from the Bohri Kadal market, district Srinagar of Kashmir valley (J&K India). Akhter H. Malik, a taxonomist at the University of Kashmir identified and verified the seeds under Voucher Specimen no. 2646-(KASH) Herbarium. For a week, the seeds were shade dried, then were ground into a coarse powder by a grinder.

### 2.2 Preparation of plant extracts

Extraction was done by successive cold extraction method using methanol as solvent. Methanol was chosen as the extraction solvent for its proven capacity to solubilize phenolic and antimicrobial compounds, its widespread use in phytochemical studies of Portulaca species, and its ability to penetrate cell matrices for efficient metabolite release. An accurately weighed 4 kg of dried (P.O) seed powder was taken in a large macerating flask and sufficient amount of extracting solvent was added to it. The flask was then kept aside for a duration of 24 h with intermittent shaking. The mixture was strained once the soluble material had been dissolved, and the marc was then pressed. Filtration was used to further clarify the combing liquid. The filtrate was then subjected to solvent recovery and the recovered solvent was again mixed with the marc in the flask. In each extraction scenario, this process was repeated three times. Then the ex-tracts were concentrated on a water bath maintained at a temperature of 50-55℃ until they transformed into a semi-solid state. To ensure complete drying, they were subsequently placed in a desiccator for storage.

### 2.3 Qualitative phytochemical analysis

For the detection of active phytochemicals like tannins, alkaloids, phytosterols, triterpenoids, flavonoids, cardiac glycosides etc., phytochemical screening of extract was carried out using standard methods^40^. A stock solution of 1 mg/ml of extract was made in its respective extracting solvent.

### 2.4 TLC profiling

The TLC profiling was done in a way as described by Biradar *et al*., 2013^41^. Dilute solutions of extract were made in methanol. These solutions served as sample solutions for TLC analysis. As an adsorbent, pre-coated TLC plates (Silica gel 60 F254) were employed, which were purchased from Merck India in Mumbai. Using fine capillaries, spotting of sample solutions was done on separately cut pre-coated TLC plates, at a point roughly around 1.5 cm from the lower (dipping) end of the plate and fairly above the surface of solvent system. Different solvent systems were tried, until we found a solvent system, when the spots got distinctively resolved on the plate. After the application of samples on the plates, each plate was placed in a separate TLC glass chambers, each containing a separate solvent system. After drying the plates, various detecting reagents were applied, but the most significant results were obtained with the anisaldehyde sulfuric acid reagent.

### 2.5 Antioxidant activity

The methanolic extract were subjected to antioxidant activity determination using DPPH Radical Scavenging Assay^42^ and Reducing Power Assay. This assay was done by the method as given by Sanja, S. D., *et al*. (2009). A stock solution was prepared by dissolving 10 mg of ascorbic acid in 10 ml of distilled water, resulting in a concentration of 1 mg/ml. Subsequently, various concentrations ranging from 5 to 50 μg/ml were prepared by adding distilled water to the stock solution and diluting accordingly. To prepare the test samples, the required amounts of extract were dissolved in the minimum amount of methanol for each concentration (100, 500, 1000, 2000, and 3000 μg/ml). The resulting solutions were then diluted to a final volume of 10 ml with phosphate buffer. UV-spectrophotometer was used to measure the absorbance of the solution at λ_max_ at 700 nm.

### 2.6 Nitric oxide radical scavenging assay

The Nitric oxide scavenging activity of methanolic extract was determined according to the method reported in publication by *Green et al*.,1982^43^. In this assay, 0.5 ml of various concentrations of extract (100–1000 μg/ml) were combined with 2 ml of sodium nitroprusside (SNP) (10 mM) in phosphate buffer saline (PBS, pH = 7.4). The mixture was then incubated at 37 ℃ for 60 min. Just before use, the Griess reagent was made by combining equal parts of 1 % sulphanilamide and 0.1 % naphthyl ethylene diamine dihydrochloride in 2.5 % phosphoric acid. An aliquot of 1.5 ml from each of the extract solutions, which had SNP and PBS added, was taken following incubation, and it was combined with an equivalent amount (1.5 ml) of Griess reagent; and the mixture was heated to 250 °C in the dark for 30 min. When nitric ions were diazotized with sulphanilamide and then coupled with naphthyl ethylene diamine dihydrochloride, the pink color chromophore was produced. Its absorbance was measured at 546 nm and compared to a blank sample. Gallic acid was employed as the standard in all experiments, which were carried out in triplicate for each concentration. The following formula was used to compute the percentage inhibition of the extract and standard as well as the percentage nitrite radical scavenging activity and IC_50_ of the extract and gallic acid:

$$\text{NO Scavenging activity (\textbackslash{}\%)}=\frac{\text{[(Abs. Control - Abs. Sample)]}}{\text{(Abs. Control)}}100$$

### 2.7 Total phenolic content

This was established by the Folin-Ciocalteu Assay, as reported in literature[44], [45].

#### 2.7.1 Preparation of standard stock solution of Gallic acid

Gallic acid stock solution (10 ml) at a concentration of 1 mg/ml was made in methanol. It was then used to prepare solutions for the calibration curve. From this, 1 ml was pipette out and transferred to a 10 ml volumetric flask. The volume was built up to 10 ml by adding the necessary volume of double distilled water to produce a concentration of 100 g/ml.

#### 2.7.2 Preparation of calibration curve of Gallic acid

Aliquots of 0.1 ml to 0.8 ml were transferred to a series of seven volumetric flasks, each holding 10 ml, from the aforementioned stock solution of 100 g/ml. Each flask received 2 ml of a 15 % sodium carbonate solution and 0.5 ml of Folin-Ciocalteu reagent, which were diluted in a 1:2 ratio with double-distilled water to obtain a solution with a Gallic acid concentration ranging from 2 g/ml to 8 g/ml. The remaining volume was filled with double-distilled water. As compared to a blank solution, the mixture's spectra showed the highest absorption at wavelength 718 nm. The calibration curve was generated by taking into account the absorbance measurements against their corresponding concentrations using linear least square regression analysis. The absorbances of all the solutions were measured at 718 nm against a blank solution.

#### 2.7.3 Preparation of sample solution

The Methanolic extract, weighing 25 mg, was carefully measured and transferred to a 25 ml volumetric flask containing a small amount of distilled water. The volume was then made up to 25 ml by adding the necessary volume of the distilled water, resulting in a solution with a concentration of 1 mg/ml. Then, a Whatman filter paper No. 41 was used to filter the solution. 6 ml of the filtered solution was transferred to a volumetric flask with a capacity of 10 ml. Two milliliters of 15 % sodium carbonate solution and 0.5 ml of the Folin-Ciocalteu reagent were added to it, along with 10 ml of double-distilled water to correct the volume. At 718 nm, the absorbance was measured in comparison to a blank solution.

### 2.8 Antibacterial activity

Bacterial strains acquired from the Microbial Type Culture Collection (MTCC) at the Institute of Microbial Technology (IMTECH) in Chandigarh, India, were used to assess the antibacterial activity of (P.O) seed extracts throughout the evaluation. *Escherchia coli*: (MTCC739), *Proteus vulgaris*: (MTCC426), *Pseudomonas aeruginosa*: (MTCC1688), *Klebsiella pneumoniae*: (MTCC432), *Salmonella typhi*: (MTCC98) *Staphylococcus aureus*: (MTCC96). Antibacterial activity of the extract was evaluated by Agar Well Diffusion Method^46^.

**Assay medium used:** Muller Hinton Agar (MHA) Medium was made by combining 38 g of the agar with 1000 ml of distilled water, then heating the mixture to boiling to completely dissolve the agar. The medium was then autoclaved for 15 min at 121 °C and 15 lbs of pressure. The autoclaved media was cooled to 45–50 °C, thoroughly mixed, and then poured (20–25 ml) onto 100 mm Petri plates.

Agar Well Diffusion Method 25 was used to investigate the antibacterial activity of the methanolic extract with few modifications. The bacterial strains were suspended in sterile water and adjusted to a turbidity of 0.5 MacFarland standard (108 CFU/ml) after being cultivated on nutrient agar at 37 °C for 18 h. By using a UV spectrophotometer set to 625 nm, turbidity was confirmed and each agar media filled petri plate was inoculated with the same suspension. Wells (diameter 8 mm) were punched in the agar and filled with 80 μl of each prepared concentration (40 mg/ml, 60 mg/ml, 80 mg/ml, 100 mg/ml) of each extract. As a positive control, streptomycin discs (25 g/disc) were positioned in the middle of each plate, along with 100 % dimethyl sulfoxide placed into a separate well in each petri plate, serving as the negative control. All the petri plates were loaded and then set aside in the laminar hood for 30 min. The petri plates were then placed in an incubator and incubated at 37 °C for 18 to 24 h before being checked for zones of inhibition. The antibacterial potential was tested by measuring the widths (in millimeters) of zones of inhibition with the help of a standard measuring scale^46^.

### 2.9 Antifungal activity

The fungal strains *Penicillium chrysogenum*: (MTCC947), *Aspergillus fumigatus*: (MTCC426), *Saccharomyces cerevisiae*: (MTCC170) procured from (IMTECH) Chandigarh, India; were used to evaluate the antifungal activity of the extract of (P.O), in addition to two strains *viz*. *Mucur albicans* and *Hypochrea viridescens*, which were procured from Soil Isolated Type Culture Collection (SITCC)-CORD, Srinagar, India. Besides these, one more strain *viz*. *Trichoderma atroviride* (MK273555) was also used^47^.

#### 2.9.1 Assay medium used

The Potato Dextrose Agar (PDA) Medium was created by combining 39 g of Potato Dextrose Agar with 1000 ml of distilled water. The mixture was then heated to boiling to ensure that all of the agar was dissolved. After that, it was autoclaved for 15 min at 121 °C and 15 lbs of pressure to sterilise it. Be thoroughly blended before using.

Agar Well Diffusion Assay 26 was used to assess the anti-fungal activity of the three (P.O) extracts with minimal changes. The fungi strains were adjusted to a turbidity of 0.5 MacFarland standard (108 CFU/ml) and suspended in sterile water. The fungi were cultivated on nutrient agar at 37 °C for 18 h. By using a UV spectrophotometer set to 625 nm, turbidity was confirmed. To inoculate petri plates with a 90 mm diameter, the suspension was utilized. Wells (diameter 8 mm) were punched in the agar and filled with 80 μl of each prepared concentration (40 mg/ml, 60 mg/ml, 80 mg/ml, 100 mg/ml) of each extract. Nystatin solution (40 µg/ml) was poured at the center well of each plate, which served as the positive control, whereas 100 % Dimethyl sulphoxide functioned as the negative control. All the petri plates were loaded and then set aside in the laminar hood for 30 min. The petri plates were then placed in an incubator and incubated at 28 °C for 18 to 24 h before being checked for zones of inhibition. With the aid of a standard measuring scale, the diameters (in millimeters) of the zones of inhibition were measured to assess the antifungal potential.

### 2.10 GC–MS profiling

The methanolic extract of the (P.O) plant was analyzed using Gas chromatography-mass spectrometry (GC–MS). The Gas Chromatography run time was 21.76 min to determine the chemical composition of the extract. The mass-spectrophotometric detector was operated in electron impact ionization mode with an ionizing energy of 75 eV to scan from *m*/*z* 50-700^48^. The NIST (National Institute of Standards and Technology) mass spectral databases were utilized to identify the peaks separated by GC–MS. The plant extract composition was determined by comparing the relative retention time (Rt) and mass spectra, enabling the identification of the components, including their names, molecular weights, and structures.

### 2.11 Computational study

#### 2.11.1 Molecular docking

For this study, we have selected various bacterial and fungal target proteins such as dihydropteroate synthase (PDB ID: 5U0V), penicillin-binding protein (PDB ID: 3MZF), beta-tubulin (PDB ID: 5FNV) and ABC transporter (PDB ID: 6J9W). Each of the four proteins fulfils a crucial function in the life cycle of both bacteria and fungi. Our selection of these protein targets is based on the specific roles they play. The X-ray crystal structure of these biomolecules were obtained from the RCSB PDB database and extracted as a PDB file. Utilizing the open-source software AutoDock 4.2^49^, we conducted molecular docking analysis, which involved tasks such as protein preparation, ligand preparation, grid generation, and the actual docking process. Subsequently, each of these biomolecules was individually input into the AutoDock molecular docking software. Protein preparation was carried out which included the removal of water molecules, elimination of redundant chains or heteroatoms, introduction of hydrogen, charge estimation, and conversion to a pdbqt file. For improved interactions, only polar hydrogens were incorporated into the protein during the preparation, and Kollman charges were added afterward. The 3-dimensional and 2-dimensional structures of the ligands Colchicine, n-decanoic acid, and triepoxydecane, were obtained using the PubChem repository. The three-dimensional structures of all the ligands were minimized in Avogadro using the UFF force field and the steepest descent algorithm. Gasteiger charges were added to the ligands in the Autodock software. Molecular docking was conducted using a grid box dimension 40 × 40 × 40 Å. Designing grid boxes with precise dimensions and a 0.3 Å spacing was a necessary step. The validation of the docking protocol was confirmed through a process involving the elimination of the inhibitor from the complex, subsequent re-docking, and the computation of the Root Mean Square Deviation (RMSD). Similar chemical interactions were noted, and it demonstrated a precise overlay with its bioactive crystallized conformation. Biovia Discovery Studio was employed to examine the interacting amino acids and the docked poses of the complex structures. The RMSD values for all three compounds were found to be below 2 Å, indicating a high level of accuracy in reproducing the experimentally determined binding poses and confirming the reliability of the docking method[50], [51], [52], [53], [54], [55], [56], [57].

#### 2.11.2 ADMET studies

The drug-likeness and predicted pharmacokinetic properties (ADMET) were evaluated using the SwissADME and pkCSM online platforms[58], [59]. The protocol for evaluating drug-likeness and pharmacokinetic properties involved submitting the chemical structures of the compounds, in SMILES format, to the SwissADME and pkCSM online platforms. SwissADME was used to assess drug-likeness based on Lipinski’s rule of five, bioavailability scores, and physicochemical properties such as molecular weight, logP, and hydrogen bond donors/acceptors. Additionally, SwissADME provided predictions for gastrointestinal absorption and blood–brain barrier permeability. The pkCSM platform complemented these analyses by predicting ADMET parameters, including absorption (e.g., Caco-2 permeability), distribution (e.g., volume of distribution), metabolism (e.g., CYP450 inhibition), excretion (e.g., renal clearance), and toxicity (e.g., AMES mutagenicity and hERG inhibition).

## 3 Results

### 3.1 Qualitative phytochemical analysis

Phytochemical analysis of the (P.O*)* seed methanolic extract revealed the presence of bioactive constituents like tannins, terpenoids, flavonoids, alkaloids, phenols, and saponins **(**Table 1**)**.

**Table 1:** Phytochemical test results of Methanolic extract of (P.O) seeds.

Class of Compounds	Phytochemical Tests	Methanolic Extract
Alkaloids	Drangendroff’s test	+
	Hager’s test	+
	Wagner’s test	+
	Mayer’s test	+

Triterpenoids	Salkowski test	+
Phytosterols	Libermann Buchard’s test	+

Flavonoids	Shinoda test	+
	Alkaline reagent test	+
	Lead acetate test	−

Saponins	Foam test	+
	Olive oil test	+
Cardiac Glycosides	Keller killani test	+
Anthraquinone Glycosides	Hydroxyanthraquinone test	−
Phenolic Compounds	Test	+
Proteins	Biuret test	+

Amino acids	Millon’s test	−
	Ninhydrin test	+

Carbohydrates	Molish’s test	+
	Barfoed’s test	−
	Seliwanoff’s test	+

Tannins	Ferric chloride test	+
	Gelatin test	+
Fats and Fixed Oils	Test	−

### 3.2 TLC profiling

**Fig. 1:**
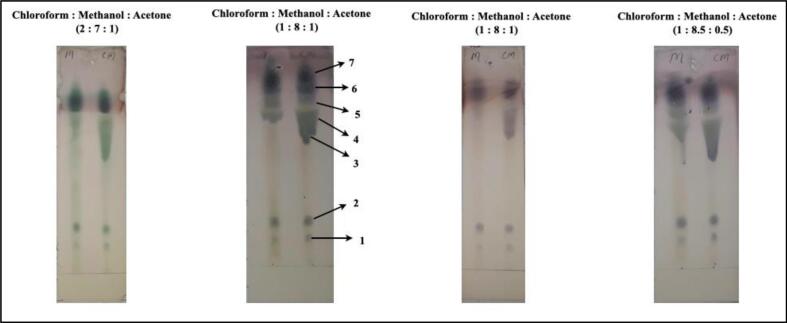
Developed TLC plates of Methanolic extract of (P.O) seeds in various solvent systems.

TLC profiling of methanolic extracts (Fig. 1) showed most of the prominent spots towards the solvent front, in the polar solvent system: chloroform: Methanol: Acetone; bearing Methanol percentage > 70 %.

### 3.3 Antioxidant activity

#### 3.3.1 DPPH radical scavenging assay

**Fig. 2:**
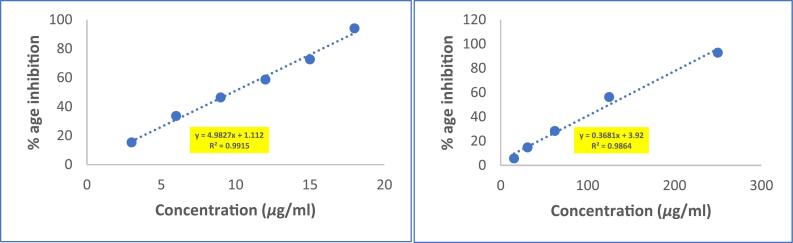
DPPH RSA of Ascorbic Acid and methanolic extract of (P.O) seeds.

The DPPH radical scavenging activity of the methanolic extract was weak as compared to that of the Ascorbic Acid with the IC_50_ values of the extract being 125.2 µg/ml and only 9.81 µg/ml in case of Ascorbic acid **(**Fig. 2**).** Multiple reasons can account for this, one of the most probable and justifying reasons might be that seeds remain in dormancy state for pretty long time and are well protected from the surroundings by impermeable seed covers, compared to other parts of the plant like leaves, stems etc. So, they don’t need that quantity of radical scavengers because radicals are not produced with that pace.

#### 3.3.2 Reducing power assay

**Fig. 3:**
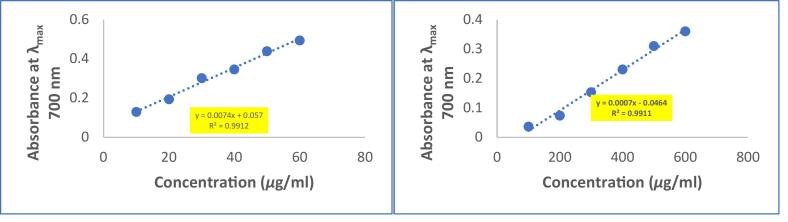
Reducing power of Ascorbic acid and methanolic extract of (P.O) seeds.

The study used a spectrophotometric method to determine the reducing ability of the methanolic extract by measuring the reduction of potassium ferricyanide (Fe^3+^) to potassium ferrocyanide (Fe^2+^) **(**Fig. 3**)**. The potassium ferrocyanide then reacted with ferric chloride to form a ferric-ferrous complex, which produced a blue color known as Perl's Prussian blue and had its highest absorption at 700 nm. The study compared the reducing power of extract with ascorbic acid and found that the extract had higher reducing power than ascorbic acid. Free radicals, produced during normal metabolic processes and other environmental conditions lack an electron and so are very reactive to get it from somewhere to become stable.

### 3.4 Nitric oxide radical scavenging assay

**Fig. 4:**
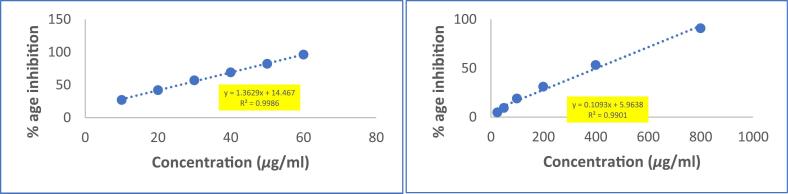
Nitric oxide scavenging activity of ascorbic acid and methanolic extract of (P.O) seeds.

Overproduction of NO radicals has been linked to several diseases. The quantity of nitric oxide can be determined using Griess reagent. When a scavenging test compound is present, the level of nitrous acid reduces, which can be measured at 546 nm. The methanolic extract demonstrated stronger nitric oxide scavenging activity (with an IC_50_ value of 402.89 μg/ml) **(**Fig. 4**)**. The scavengers present in extract were able to scavenge NO radicals in a manner that was dependent on the dose, indicating a gradual increase in the scavenging activity as the dose was increased.

### 3.5 Antimicrobial activity

**Fig. 5:**
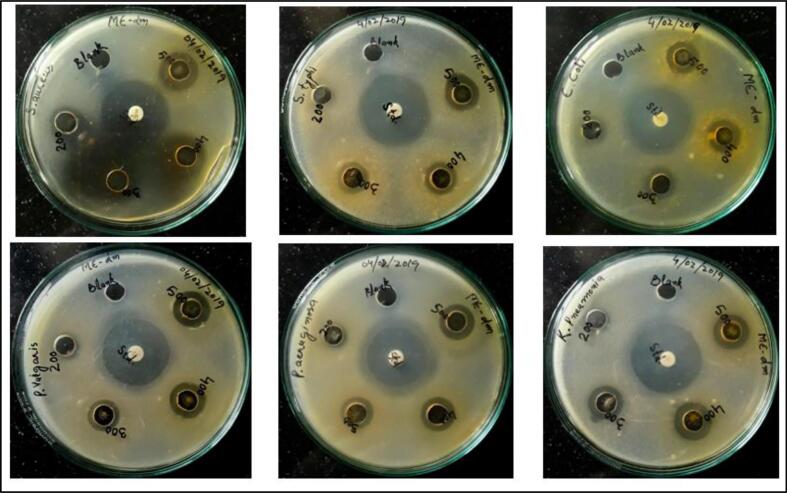
Photographs of antibacterial activity of (P.O) seeds methanolic extract.

**Fig. 6:**
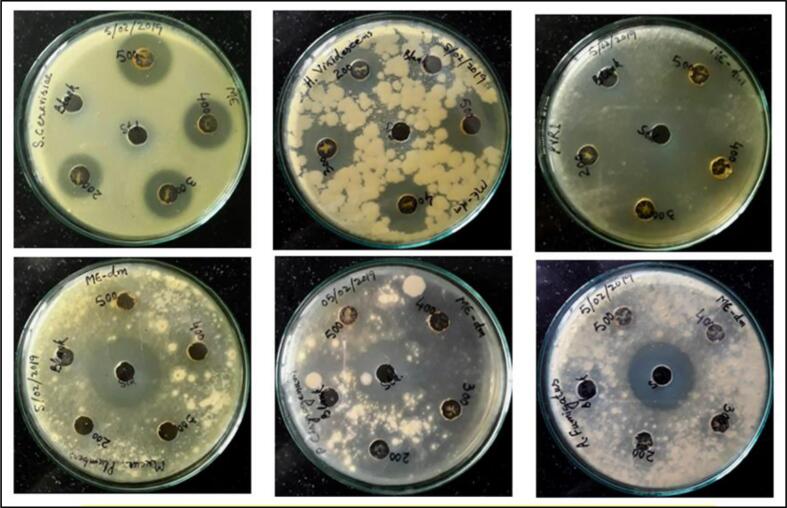
Photographs of antifungal activity of (P.O) seeds methanolic extract.

The antibacterial and antifungal results of methanolic extract of (P.O) seeds and controls (positive & negative) are presented in **(**Table 2, Table 3**) and (**Fig. 5, Fig. 6**)**. Except two fungi, the methanolic extract actively inhibited the growth of all tested bacteria and fungi, particularly at the higher doses. Although it did show activity at lower doses also, the zones of inhibition were less defined and clear. At higher doses, it showed clear and transparent zones of inhibition against all the test bacteria and at all the doses used. In case of fungi, two strains were tremendously inhibited at all doses, while as the zones of inhibition could not be demarcated in other fungal strains used. There was not a proper pattern of increase in the diameters of zones of inhibition, but a general increase was observed with increasing dose. The most probable reason for this might be the improper distribution of either inoculum or media in the plates. The methanolic extract showed almost equal potency against all the tested bacteria except the *Staphylococcus aureus*, which was somewhat more sensitive to methanolic extract and showed 16 mm inhibition zones across 60–100 mg/ml. The fungal strains inhibited by methanolic extract included *Saccharomyces cerevisiae, Hypochrea* viridescens and *Trichoderma atroviride* showing inhibition zones of 23 mm, 23 mm and 20 mm at 100 mg/ml respectively.

**Table 2:** Diameters of antibacterial zones of inhibition of (P.O) seeds of methanolic extract.

Bacterial strain	Diameters of zones of inhibition (in mm)					
	**Methanolic extract concentrations**				**Positive control**	**Negative control**
	100 mg/ml	80 mg/ml	60 mg/ml	40 mg/ml	Streptomycin (25 µg/disk)	DMSO
*Staphylococcus aureus* (MTCC96)	16 ± 0.577	16 ± 0.577	16 ± 1.00	13 ± 0.577	28 ± 1.00	−
*Salmonella typhi* (MTCC98)	15 ± 1.00	13 ± 1.00	12 ± 0.577	11 ± 0.577	29 ± 1.00	−
*Escherchia coli* (MTCC739)	15 ± 0.577	14 ± 1.00	13 ± 0.577	12 ± 1.00	28 ± 0.577	−
*Proteus vulgaris* *(*MTCC426)	16 ± 1.00	15 ± 1.00	13 ± 0.577	11 ± 1.00	28 ± 1.00	−
*Pseudomonas aeruginosa* (MTCC1688)	16 ± 0.577	15 ± 0.577	14 ± 0.577	10 ± 1.00	26 ± 1.00	−
*Klebsiella pneumoniae* (MTCC432)	15 ± 0.577	15 ± 0.577	14 ± 0.577	10 ± 1.00	29 ± 0.577	−

**Table 3:** Diameters of anti-fungal zones of inhibition of (P.O) seeds Methanolic extract.

Fungal strain	Diameters of zones of inhibition (mm)					
	**Methanolic’ extract concentrations**				**Positive control**	**Negative control**
	100 mg/ml	80 mg/ml	60 mg/ml	40 mg/ml	Nystatin (40 µg/ml)	DMSO (10 %)
*Penicillium* *chrysogenum* (MTCC947)	−	−	−	−	26 ± 0.577	−
*Aspergillus fumigatus* (MTCC426)	−	−	−	−	29 ± 0.577	−
*Saccharomyces cerevisiae* (MTCC170)	23 ± 0.577	20 ± 0.577	19 ± 0.577	18 ± 0.577	−	−
*Mucor plumbeus* (SITCC)-CORD	−	−	−	−	30 ± 0.577	−
*Hypochrea viridescens* (SITCC)-CORD	23 ± 0.577	21 ± 0.577	20 ± 1.00	19 ± 1.00	−	−
*Trichoderma atroviride* (MK273555)	20 ± 0.577	18 ± 1.00	17 ± 1.00	18 ± 0.577	28 ± 0.577	−

As the Positive Control *Streptomycin* was active against all the tested bacteria, so was the methanolic extract, thereby suggesting that it contains antimicrobials which might be acting by a similar mechanism of action as Streptomycin. Streptomycin inhibits the process of protein synthesis by binding to the 16S rRNA present in the smaller subunit of the bacterial ribosome. This binding disrupts the attachment of formyl-methionyl-tRNA to the 30S subunit, thereby interfering with the synthesis of proteins in the bacterial cell. To put it simply, streptomycin prevents the bacterial ribosome from making proteins by blocking the attachment of certain molecules. There are reports of *Saccharomyces cerevisiae*, involved in diseases like vaginal infections, fungemia etc. So methanolic’ extract or the antifungals present in the extract can prove a better choice in these situations.

### 3.6 GC–MS profiling

**Fig. 7:**
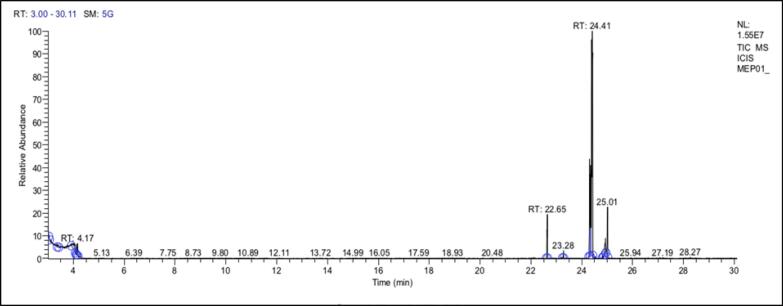
GC–MS Chromatogram of methanolic extract of (P.O).

The methanolic extract of (P.O) seeds was analyzed using GC–MS chromatography, which revealed 12 major peaks representing different phytochemical constituents (Fig. 7, Table 4). The peaks were identified using the NIST mass spectral databases by comparing their relative retention time (R*_t_*) and mass spectra. This identification method helped to determine the components present in the extract. The molecular basis for the anti-bacterial and antifungal activity displayed by Colchicine, n-decanoic acid, and triepoxydecane was explored using an *in-silico* molecular docking approach. The findings suggest that all three ligands exhibit superior interactions with fungal targets in comparison to bacterial targets. Notably, colchicine exhibited superior binding affinity with beta-tubulin (5FNV) and ABC transporter (6J9W), suggesting that it may show potential antifungal activity through binding with these specific protein targets.

**Table 4:** Identified phytochemicals of Methanolic extract of Portulaca oleracea seeds.

**S.No**	**R.T. (min)**	**Peak height**	**Peak area**	**Area (%)**	**Molecular formula**	**Name**
1	3.06	163825.33	140655.49	0.18	C_22_H_25_NO_6_	Colchicine
2	3.4	159379.96	457972.62	0.60	C_14_H_32_P_2_Ru	Ruthenium, bis[(1,2,3-ü)-2-methyl-2-propenyl]bis(trimethylpho sphine)-
3	4.04	545256.60	2859113.55	3.37	C_30_H_50_O_6_	Olean-12-ene-3,15,16,21,22,28-hexol, (3á,15à,16à,21á,22à)-
4	4.13	737828.79	832227.34	1.08	C_35_H_46_O_8_	2,4,6-Decatrienoic acid, 1a,2,5,5a,6,9,10,10a-octahydro-5,5a-dihydroxy-4-(h ydroxymethyl)-1,7,9-trimethyl-1-[[(2-methyl-1-oxo −2-butenyl)oxy]methyl]-11-oxo-1H-2,8a-methanocy clopenta[a]cyclopropa[e]cyclodecen-6-yl ester
5	4.17	841783.67	1120679.96	1.46	C_20_H_24_O_5_	Gibbane-1,10-dicarboxylic acid, 2,3-epoxy-4a-hydroxy-1-methyl-8-methylene-, 1,4a-lactone, 10-methyl ester, (1à,2á,3á,4aà,4bá,10á)-
6	22.65	2973486.54	3946534.58	5.14	C_17_H_32_O_2_	Cyclopentaneundecanoic acid, methyl ester
7	23.28	504063.87	587529.17	0.77	C_10_H_20_O_2_	n-Decanoic acid
8	24.31	6646835.43	11762224.28	15.33	C_13_H_24_O	11-Tridecyn-1-ol
9	24.41	15262885.89	47214809.80	61.54	C_10_H_16_O_3_	1,2:4,5:9,10-Triepoxydecane
10	24.82	184703.01	190832.59	0.25	C_10_H_20_O	9-Decen-1-ol
11	24.93	1046775.30	2357662.98	3.07	C_13_H_24_O	11-Tridecyn-1-ol
12	25.01	3348380.90	5257991.72	6.85	C_18_H_30_O	9,12,15-Octadecatrienal

### 3.7 Molecular docking studies

The molecular basis for the anti-bacterial and antifungal activity displayed by Colchicine, n-decanoic acid, and triepoxydecane was explored using an *in-silico* molecular docking approach. In antibacterial and antifungal drug development, various molecular targets are explored to disrupt specific biological processes essential for the survival and replication of bacteria and fungi. Several typical molecular targets have emerged for research and drug development include dihydropteroate synthase (PDB ID: 5U0V), penicillin-binding protein (PDB ID: 3MZF), beta-tubulin (PDB ID: 5FNV) and ABC transporter (PDB ID: 6J9W). Dihydropteroate synthase (DHPS) is a protein target commonly associated with antibacterial drug development. DHPS is involved in the folate biosynthesis pathway, a crucial pathway for the synthesis of purines and pyrimidines, essential components of DNA. Penicillin-binding proteins (PBPs) are a crucial molecular target for antibiotics and play a vital role in bacterial cell wall synthesis. Beta-tubulin is a key molecular target in the context of antifungal and anticancer drug development. Tubulins are structural proteins that polymerize to form microtubules, essential components of the cytoskeleton in eukaryotic cell. Similarly, ABC transporters can indeed be an antifungal target, specifically in addressing resistance mechanisms.

**Fig. 8:**
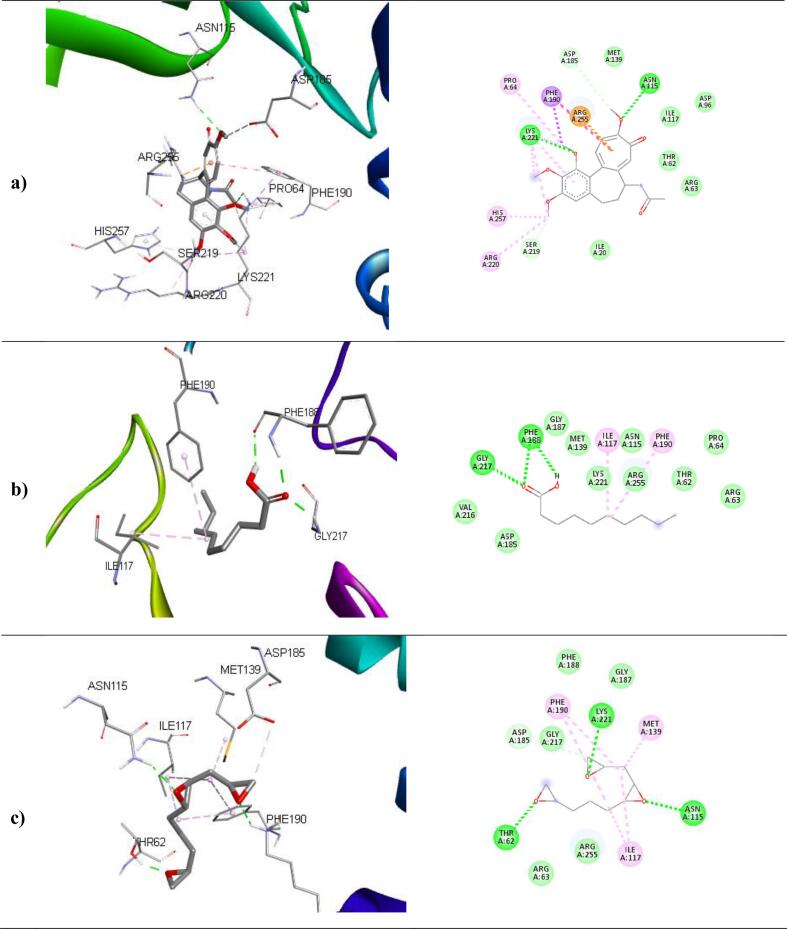
3D and 2D Molecular level interactions of a) colchicine b) n-decanoic acid and c) triepoxydecane with the binding site residues of dihydropteroate synthase (PDB ID: 5U0V).

**Fig. 9:**
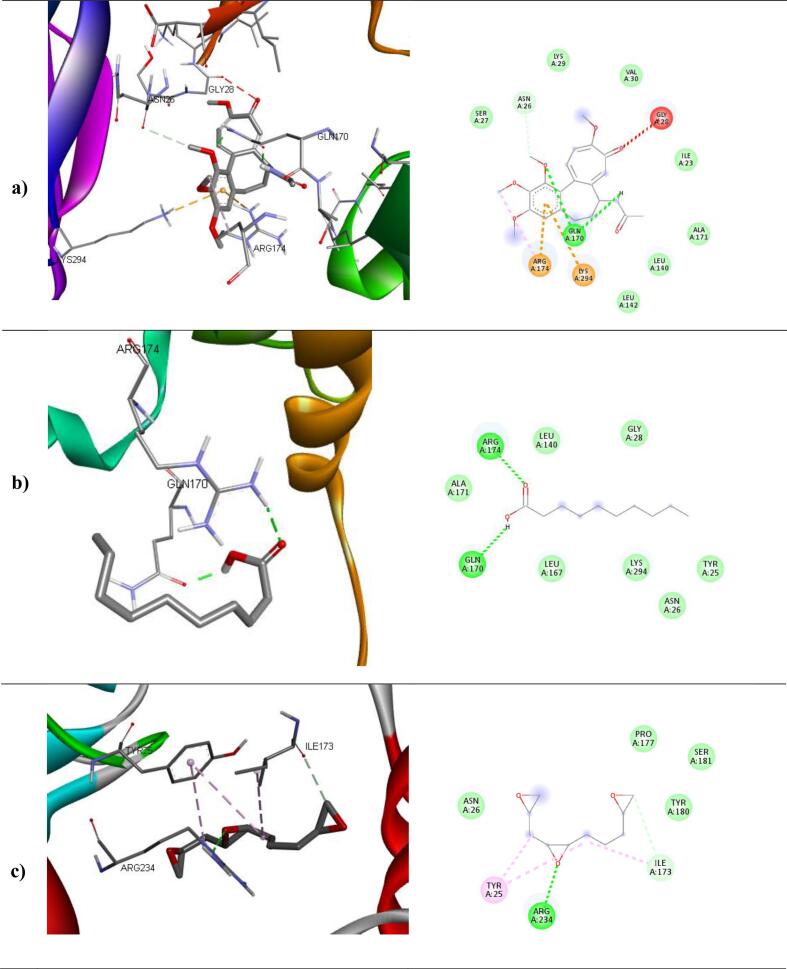
3D and 2D Molecular level interactions of a) colchicine b) n-decanoic acid and c) triepoxydecane with the binding site residues of penicillin-binding protein (PDB ID: 3MZF).

**Fig. 10:**
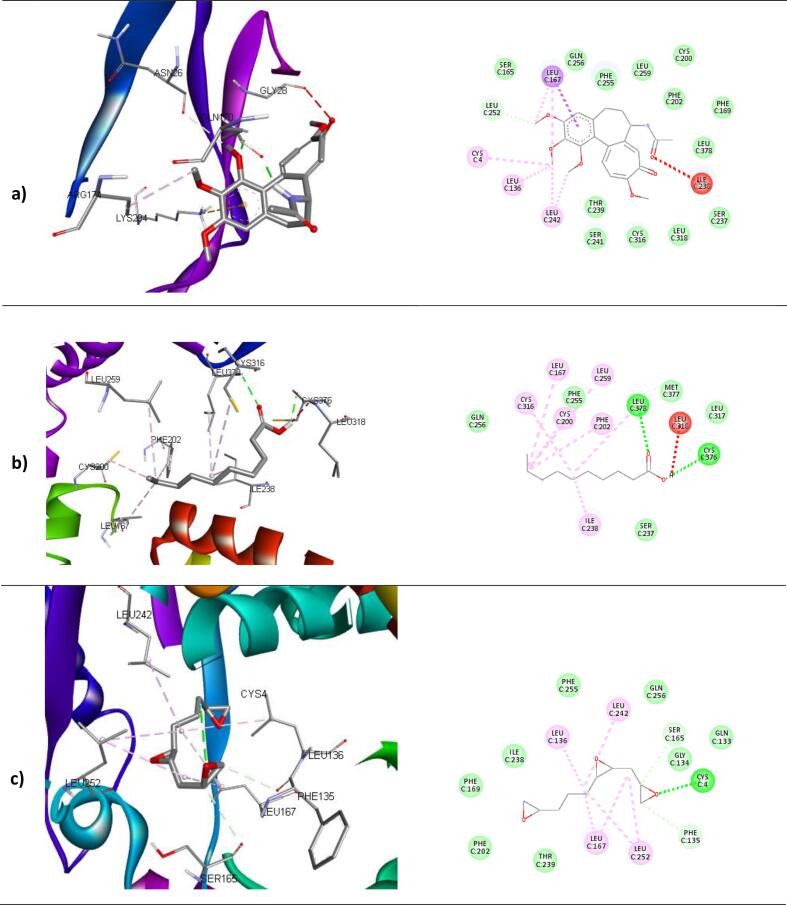
3D and 2D Molecular level interactions of a) colchicine b) n-decanoic acid and c) triepoxydecane with the binding site residues of beta-tubulin (PDB ID: 5FNV).

**Fig. 11:**
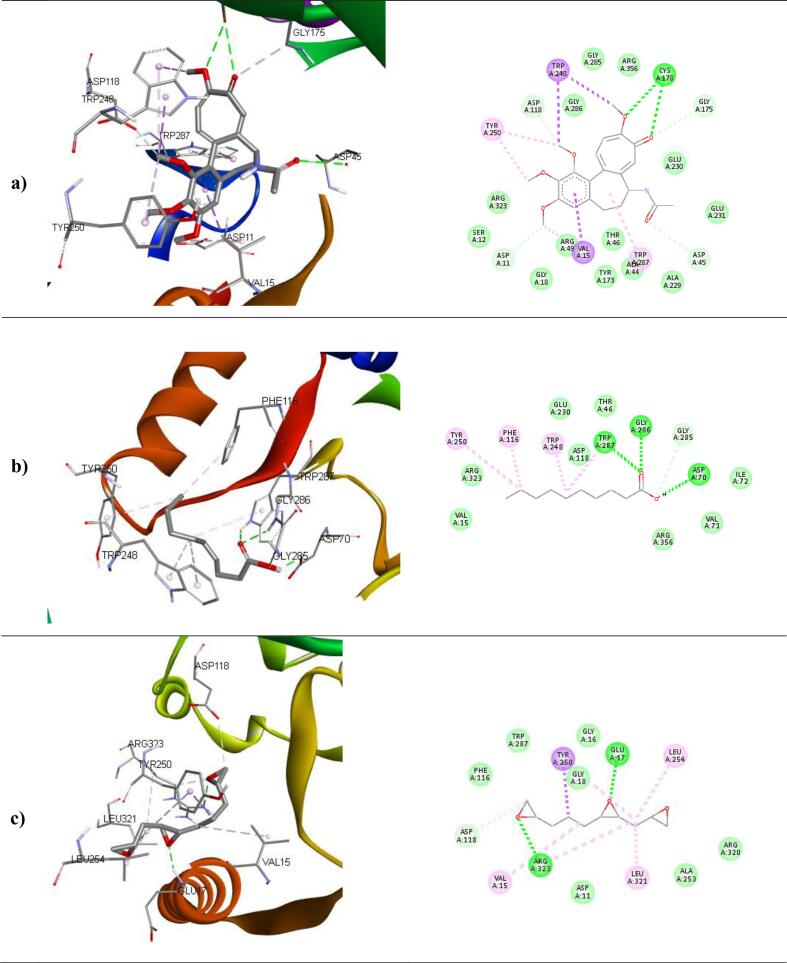
3D and 2D Molecular level interactions of a) colchicine b) n-decanoic acid and c) triepoxy decane with the binding site residues of ABC transporter (PDB ID: 6J9W).

The binding affinities and corresponding important interacting residues are shown in **(**Table 5**)**. 3D and 2D Molecular level interactions of a) colchicine b) n-decanoic acid and c) triepoxydecane with the binding site residues of dihydropteroate synthase (PDB ID: 5U0V), penicillin-binding protein (PDB ID: 3MZF), beta-tubulin (PDB ID: 5FNV) and ABC transporter (PDB ID: 6J9W) are shown in **(**Fig. 8, Fig. 9, Fig. 10, Fig. 11 **)**, respectively. In the context of bacterial targets, Triepoxydecane demonstrated a more favorable docking score of −6.40 kcal/mol against dihydropteroate synthase (5U0V), while colchicine exhibited a docking score of −6.54 kcal/mol against penicillin-binding protein (3MZF). Since triepoxydecane possess three epoxy rings which have a hydrogen bond accepting capability, it exhibited hydrogen bonding interactions with THR62, ASN115 and LYS221 residues of dihydropteroate synthase apart from other interactions with ILE117, MET139, ASP185 and PHE190 as shown in. Colchicine displayed hydrogen bonding interaction with GLN170, and additionally, pi-cation interactions were observed between the phenyl ring of colchicine and the ARG174 and LYS294 residues of the target protein, namely penicillin-binding protein as shown in. In case of fungal targets, colchicine showed a better binding affinity of −9.77 kcal/mol with both the targets i.e., beta-tubulin (5FNV) and ABC transporter (6J9W) as shown in Table 5. Colchicine interacted with beta-tubulin (5FNV) protein mainly via Vander Waal forces with significant amino acids such as SER165, PHE169, PHE202, SER237, PHE255, CYS316 and LEU318. Other interactions include π-alkyl interactions with CYS4, LEU136 and LEU242. Colchicine interacted with ABC transporter (6J9W) protein mainly via hydrogen bonding and Vander Waal forces. A hydrogen bond interaction was observed between the cycloheptatrienone ring of colchicine and the CYS 178 residue of the target protein. Vander Waal interactions were also noted with SER12, GLY285, GLY286, ARG323 and ARG356 residues of the target protein. In summary, the findings suggest that all three ligands exhibit superior interactions with fungal targets in comparison to bacterial targets. Notably, colchicine exhibited superior binding affinity with beta-tubulin (5FNV) and ABC transporter (6J9W), suggesting that it may show potential antifungal activity through binding with these specific protein targets.

**Table 5:** The binding affinity of selected compounds against microbial proteins.

Target Protein (PDB ID)	Binding affinity (kcal/mol)	Ligand	Interacting Residues
5U0V	−4.65	Colchicine	PRO64, ASN115, PHE190, SER219, LYS221, ARG 255
	−5.89	n-decanoic acid	ILE117, PHE188, PHE190, GLY217
	**−6.40**	Triepoxydecane	THR62, ASN115, ILE117, MET139, ASP185, PHE190, LYS221

3MZF	**−6.54**	Colchicine	ASN26, GLN170, ARG174, LYS294
	−3.56	n-decanoic acid	GLN170, ARG174
	−4.67	Triepoxydecane	TYR25, ILE173, ARG234

5FNV	**−9.77**	Colchicine	CYS4, LEU136, LEU167, LEU242, LEU252
	−5.11	n-decanoic acid	LEU167, CYS200, LEU259, CYS316, CYC376, LEU378
	−6.34	Triepoxydecane	CYS4, LEU136, PHE135, LEU167, LEU242, LEU252

6J9W	**−9.77**	Colchicine	VAL15, ASP11, ARG49, CYS178, TRP248, TYR250
	−6.16	n-decanoic acid	ASP70, PHE116, TRP248, TYR250, TRP287, GLY286
	−7.58	Triepoxydecane	GLU17, GLY18, ASP118, TYR250, LEU254, ARG323

### 3.8 ADMET studies

An *in silico* evaluation of drug-likeness and ADMET properties was performed for the selected compounds i.e., Colchicine, n-Decanoic acid, and Triepoxydecane, as presented in Table 6, Table 7. This analysis aimed to predict their pharmacokinetic behaviour and overall drug-like characteristics, which are critical for determining their potential as therapeutic agents. Lipinski's Rule of Five (Ro5) assesses key molecular features that influence oral bioavailability, suggesting a compound is more likely to be orally active if it has no more than one violation of the following criteria: no more than 5 hydrogen bond donors, no more than 10 hydrogen bond acceptors, a molecular weight under 500 Daltons, and a LogP value not exceeding 5. All the three compounds adhered to Lipinski’s rule and demonstrated favourable drug-likeness, including the ability to cross the blood–brain barrier. The ADMET properties were predicted to evaluate the pharmacokinetic profile of each compound. Absorption analysis included predictions of intestinal uptake and bioavailability. Distribution assessments focused on the ability to cross the blood–brain barrier and bind to plasma proteins. Metabolic profiling examined potential interactions with cytochrome P450 enzymes. Excretion studies explored possible elimination routes and rates, while toxicity evaluations aimed to identify potential adverse effects and assess overall safety. This comprehensive *in silico* analysis provided an initial overview of each compound's pharmacokinetic behaviour, offering valuable insight into their potential as safe and effective therapeutic agents. Our study revealed important insights into the oral bioavailability (OB) and intestinal absorption of the selected compounds, with all tested molecules showing favourable outcomes in these aspects. Caco-2 permeability values, which serve as indicators of absorption potential, were recorded as follows: Colchicine − 1.139, n-Decanoic acid − 1.564, and Triepoxydecane − 1.370. Notably, higher Caco-2 permeability is associated with improved intestinal absorption. Further pharmacokinetic analysis included predictions of volume of distribution at steady state (Vdss), blood–brain barrier (BBB) permeability, and central nervous system (CNS) penetration. The Vdss values (log L/kg) were: Colchicine – −0.314, n-Decanoic acid – −0.697, and Triepoxydecane − 0.175. For BBB permeability (log BB), the results were: Colchicine – −0.712, n-Decanoic acid – 0.142, and Triepoxydecane – −0.099. CNS permeability (log PS) was found to be: Colchicine – 3.091, n-Decanoic acid – 2.143, and Triepoxydecane – 2.975. These findings suggest that all compounds exhibit promising potential for crossing the BBB. In terms of elimination, total drug clearance was assessed to help predict the likelihood of toxicity. The clearance values were: Colchicine – 0.227, n-Decanoic acid – 1.552, and Triepoxydecane – 1.24. Lastly, genotoxicity was evaluated using the Ames test. Colchicine and n-Decanoic acid tested negative for mutagenicity, indicating no Ames toxicity, while Triepoxydecane showed a positive result.Table 8..

**Table 6:** Drug-likeness property prediction of selected compounds.

**Compound**	**MW**	**mLogP**	**HBA**	**HBD**	**TPSA**	**Lipinski's Rule (Ro5)**
**Colchicine**	399.44	1.02	6	1	83.09	Yes
**n-Decanoic acid**	172.26	2.58	2	1	37.30	Yes
**Triepoxydecane**	184. 23	0.64	3	0	37.59	Yes

MW: molecular weight; mLogP: lipophilicity; HBA: hydrogen bond acceptor; HBD: hydrogen bond donor; TPSA: topological polar surface area.

**Table 7:** *In silico* Absorption and Distribution prediction of selected compounds.

**Compound**	**Absorption**		**Distribution**		
	**Caco2** **Permeability**	**Intestinal absorption** **(human)**	**VDss** **(human)**	**BBB permeability**	**CNS** **permeability**
	**Numeric (log Papp in 10^-6^ cm/s)**	**Numeric (%absorbed)**	**Numeric** **(log L kg^−1^)**	**Numeric** **(log BB)**	**Numeric** **(log PS)**
Colchicine	1.139	97.245	−0.314	−0.712	−3.091
n-Decanoic acid	1.564	94.066	−0.697	0.142	−2.143
Triepoxydecane	1.370	100	0.175	−0.099	−2.975

**Table 8:** *In silico* Metabolism, Excretion and Toxicity prediction of selected compounds.

Compound	Metabolism							Excretion	Toxicity
	**Substrate**		**Inhibitor**					**Total clearance**	**AMES toxicity**
	**CYP**								
	**2D6**	**3A4**	**1A2**	**2C19**	**2C9**	**2D6**	**3A4**	**Numeric (log mL min ^-1^ kg ^-1^)**	**Categorical (yes/no)**
	**CategoricNoal (yes/no)**								
Colchicine	No	Yes	Yes	Yes	No	No	Yes	0.227	No
n-Decanoic acid	No	No	No	No	No	No	No	1.552	No
Triepoxydecane	No	No	No	No	No	No	No	1.24	Yes

## 4 Discussion and conclusion

The methanolic extract of (P.O) seeds exhibited a rich phytochemical profile, with flavonoids, phenolics, tannins, and triterpenoids identified during preliminary screening. These findings align with prior studies on *Portulaca* species, where methanol; a polar solvent; has been shown to efficiently extract polar secondary metabolites such as flavonoids and phenolic acids due to their hydroxyl groups and hydrophilic nature[32], [48], [60], [61]. Thin-layer chromatography (TLC) further corroborated the polarity of these compounds, as higher R*_f_* values (spots near the solvent front) are consistent with the migration behavior of polar phytochemicals like phenolics in polar mobile phases. This polarity may reflect the ecological role of seeds, which often accumulate water-soluble antioxidants to counteract oxidative stress during prolonged dormancy[62], [63], [64].

The antioxidant activity of the methanolic extract was assessed through multiple assays. The DPPH radical scavenging activity (IC_50_: 125.2 µg/mL) was notably weaker than ascorbic acid (IC_50_: 9.81 µg/mL), a trend observed in other seed extracts such as *Nigella sativa* and *Linum usitatissimum*[48], [60], [61], [62], [63], [64], [65], [66]. This reduced activity may stem from the metabolic constraints of dormant seeds, which are shielded by impermeable seed coats and likely prioritize structural defenses (e.g., lignins) over soluble antioxidants[63], [67]. In contrast, the extract demonstrated superior reducing power compared to ascorbic acid, a phenomenon also reported for *Moringa oleifera* seed extracts[61], [68]. This enhanced reducing capacity could be attributed to the synergistic action of polyphenols and tannins, which donate electrons via redox-active hydroxyl groups to reduce Fe^3+^ to Fe^2+^[62], [63], [64]. The nitric oxide (NO) scavenging activity (IC_50_: 402.89 µg/mL) further highlights the extract’s bioactivity. While NO quenching by plant extracts is often linked to flavonoid-mediated neutralization of reactive nitrogen species, the moderate IC_50_ here suggests contributions from triterpenoids, which are known to inhibit nitric oxide synthase (NOS) in inflammatory pathways[69], [70]. For instance, betulinic acid, a triterpenoid, has been shown to suppress NOS expression in macrophages, a mechanism that may parallel the activity of *(P.O)oleracea* seed constituents^71^.

The methanolic extract exhibited broad-spectrum antimicrobial activity, with dose-dependent inhibition zones against both tested bacteria and fungi. The heightened sensitivity of *Staphylococcus aureus* (16 mm at 60–100 mg/ml) aligns with studies showing Gram-positive bacteria’s susceptibility to phenolic acids due to their lack of an outer membrane, unlike Gram-negative species[72], [73], [74]. However, inconsistent inhibition zones in fungal assays-such as the inability to demarcate zones in certain strains-mirrors challenges reported in other agar diffusion studies, where uneven inoculum distribution or agar thickness can skew results. The strong inhibition of *Saccharomyces cerevisiae* (23 mm at 100 mg/ml) is particularly noteworthy, due to its role as an opportunistic pathogen in fungemia. This activity may be attributed to flavonoid-induced membrane disruption, a mechanism reported for phenolic compounds in plant extracts, such as *Quercus robur* seeds against *Candida albicans*[71], [72], [75], [76]. However, inconsistent inhibition zones in fungal assays, such as the inability to demarcate zones in certain strains, reflect challenges in agar diffusion studies, where uneven inoculum distribution or agar thickness can skew results. The extract’s bactericidal potency, comparable to streptomycin, suggests ribosomal targeting similar to aminoglycosides, which block protein synthesis by binding 16S rRNA. While the exact mechanism remains unconfirmed, GCMS-identified constituents like n-decanoic acid are known to disrupt bacterial membranes, as observed in *Lactobacillus* spp.[77], [78], [79], [80]. Molecular docking of colchicine revealed strong binding affinity for fungal beta-tubulin (PDB: 5FNV) and ABC transporter (PDB: 6J9W), aligning with its established role in inhibiting microtubule assembly in eukaryotes and suggesting a targeted antifungal mechanism[81], [82], [83], [84].

As the first study to employ GCMS analysis for *Portulaca oleracea* seeds, 68 bioactive compounds were identified, including colchicine, n-decanoic acid, and triepoxydecane. The *in silico* evaluation of drug-likeness and ADMET properties for these three compounds provided insights into their therapeutic potential. All compounds adhered to Lipinski’s Rule of Five, indicating favorable drug-likeness and oral bioavailability. Key molecular properties, including molecular weight, lipophilicity, hydrogen bond donors and acceptors, and topological polar surface area, were within acceptable ranges, suggesting robust pharmacokinetic profiles. Absorption analysis revealed high intestinal absorption, with Caco-2 permeability values of 1.139, 1.564, and 1.370 for colchicine, n-decanoic acid, and triepoxydecane, respectively, and human intestinal absorption percentages ranging from 94.06 % to 100 %. Distribution predictions confirmed the compounds’ ability to cross the blood–brain barrier, with n-decanoic acid showing the highest BBB permeability (log BB: 0.142). CNS permeability values supported their potential to penetrate the central nervous system, particularly for colchicine (log PS: −3.091). Metabolic profiling indicated that colchicine interacts with cytochrome P450 enzymes (CYP3A4, CYP1A2, and CYP2C19), while n-decanoic acid and triepoxydecane showed no significant interactions. Excretion analysis revealed varying clearance rates, with n-decanoic acid exhibiting the highest (1.552 log mL min^−1^ kg^−1^). Toxicity evaluations using the Ames test showed that colchicine and n-decanoic acid were non-mutagenic, supporting their safety, whereas triepoxydecane tested positive for mutagenicity, raising concerns about its safety profile.

The study has limitations that warrant consideration. The use of methanol as a polar solvent likely excluded non-polar antimicrobials, such as essential oils, which have shown activity in other *Portulaca* studies. The agar diffusion method, while widely used, is semi-quantitative and prone to variability; broth microdilution assays (e.g., MIC/MBC) would provide more reproducible metrics. Additionally, the absence of compound isolation (e.g., via HPLC) limits mechanistic certainty, as synergistic or antagonistic interactions among co-extracted phytochemicals cannot be ruled out.

The scope of this study highlights the potential of *Portulaca oleracea*, a plant with a history of use in traditional medicine, as a source of bioactive compounds for therapeutic and agricultural applications. The favorable drug-likeness and pharmacokinetic properties of colchicine and n-decanoic acid suggest their potential in addressing clinical conditions, such as anti-inflammatory, antimicrobial, or neuroprotective applications, given their ability to cross the blood–brain barrier. The identification of 68 bioactive compounds via GCMS analysis underscores the chemical diversity of the methanolic seed extract, aligning with the growing interest in natural products for drug discovery. However, the positive Ames test result for triepoxydecane emphasizes the need for careful safety evaluations of natural product-derived compounds. The extract’s efficacy against drug-resistant pathogens like *Staphylococcus aureus* and *Saccharomyces cerevisiae* positions it as a candidate for pharmaceutical applications, such as topical antimicrobial formulations, or agricultural uses, including eco-friendly biofungicides.

Future perspectives include conducting *in vitro* and *in vivo* studies to validate the *in silico* predictions and confirm the therapeutic efficacy and safety of colchicine and n-decanoic acid, prioritizing their anti-inflammatory, antioxidant, and antimicrobial activities. For triepoxydecane, further toxicological studies are essential to elucidate its mutagenic potential and explore structural modifications to mitigate toxicity while retaining its favorable pharmacokinetics. Bioassay-guided fractionation and advanced analytical techniques, such as HPLC, should be employed to isolate and characterize active compounds, enabling mechanistic studies of their interactions with biological targets. Exploring synergistic effects among the extract’s constituents could uncover enhanced therapeutic benefits. Additionally, clinical trials to standardize dosages and *in vivo* infection models to validate efficacy are critical for translating these findings into practical applications. Expanding GCMS analysis to other parts of *Portulaca oleracea* or using different extraction solvents could identify novel bioactive compounds, broadening the plant’s therapeutic and agricultural applications. Advanced computational modeling, including molecular dynamics simulations, could further refine predictions of compound-target interactions. Testing the extract’s utility in post-harvest crop protection could also bridge traditional medicinal knowledge with scalable solutions for agricultural challenges.

## 5 Conclusion

In conclusion, the methanolic extract of *Portulaca oleracea* seeds demonstrates significant antioxidant and antimicrobial potential, driven by polar phytochemicals such as flavonoids and triterpenoids, with notable efficacy against drug-resistant pathogens. The identification of 68 bioactive compounds via GC–MS, coupled with *in silico* ADMET evaluations and molecular docking, highlights the therapeutic promise of colchicine and n-decanoic acid, while caution is warranted for triepoxydecane due to its genotoxicity. These findings position the extract as a versatile candidate for pharmaceutical and agricultural applications, bridging traditional knowledge with modern drug discovery and sustainable agriculture. By addressing methodological limitations and pursuing rigorous validation, this study lays a foundation for developing evidence-based, natural product-derived solutions to global health and agricultural challenges.

## CRediT authorship contribution statement

**Khursheed Ahmad Sheikh:** Methodology, Investigation, Formal analysis, Data curation. **Reyaz Hassan Mir:** Writing – original draft, Validation, Software, Resources. **Mohammad Ovais Dar:** Writing – review & editing, Visualization, Validation, Resources. **Adil Farooq Wali:** Writing – original draft, Software, Formal analysis, Data curation. **Insha Qadir:** Writing – original draft, Resources, Investigation, Formal analysis. **Sheeba Nazir:** Writing – original draft, Resources, Investigation, Data curation. **Mohammed Iqbal Zargar:** Visualization, Supervision, Methodology, Investigation. **Sirajunisa Talath:** Writing – review & editing, Validation, Software, Resources. **Sathvik B. Sridhar:** Writing – original draft, Visualization, Validation, Software. **Javedh Shareef:** Writing – review & editing, Software, Resources. **Mubashir Hussain Masoodi:** Validation, Supervision, Resources, Conceptualization.

## Declaration of competing interest

The authors declare that they have no known competing financial interests or personal relationships that could have appeared to influence the work reported in this paper.
